# Advances in bladder cancer imaging

**DOI:** 10.1186/1741-7015-11-104

**Published:** 2013-04-10

**Authors:** Shaista Hafeez, Robert Huddart

**Affiliations:** 1 The Royal Marsden NHS Foundation Trust and the Institute of Cancer Research, Sutton, Surrey, UK

**Keywords:** Bladder cancer, Diffusion-weighted MRI, Multidetector computed tomography, PET-CT, Staging, Ultrasound, Virtual cystoscopy

## Abstract

The purpose of this article is to review the imaging techniques that have changed and are anticipated to change bladder cancer evaluation. The use of multidetector 64-slice computed tomography (CT) and magnetic resonance imaging (MRI) remain standard staging modalities. The development of functional imaging such as dynamic contrast-enhanced MRI, diffusion-weighted MRI and positron emission tomography (PET)-CT allows characterization of tumor physiology and potential genotypic activity, to help stratify and inform future patient management. They open up the possibility of tumor mapping and individualized treatment solutions, permitting early identification of response and allowing timely change in treatment. Further validation of these methods is required however, and at present they are used in conjunction with, rather than as an alternative to, conventional imaging techniques.

## Introduction

Bladder cancer is a common disease with significant associated morbidity and mortality. Globally it is the ninth most common cause of cancer related death in men [1,2]. The clinical spectrum at presentation can be divided in to those with (i) superficial or non-muscle-invasive bladder cancer (NMIBC), approximately 70% to 80% of patients; (ii) muscle-invasive bladder cancer (MIBC), approximately 20% at presentation; and (iii) metastatic disease [3].

### The challenge

Anatomically the bladder is divided into several layers. Accurate local staging with imaging is dependent on reliably distinguishing these layers. Clinical staging of the primary tumor is with bimanual examination under anesthesia. In those with MIBC this has been shown to be inaccurate in 23% to 50% of cases [4,5]. Transurethral resection of bladder tumor (TURBT) also understages tumors; approximately 30% to 50% patients are understaged at the time of cystectomy [6-10]. Therefore, accurate radiological correlation is important to help guide patient management.

The objective of any staging modality is to achieve adequate visualization of the primary tumor, extent of loco regional disease and to determine the presence of metastases. However, conventional imaging modalities are unable to identify microscopic disease and can be inaccurate in identifying macroscopic disease. To address this newer imaging techniques are being explored to improve pretreatment staging, predict early response to treatment and provide non-invasive alternatives to cystoscopy for those requiring long-term surveillance. This article will review the development of imaging techniques that have changed, and are anticipated to change, bladder cancer evaluation.

### Computed tomography (CT)

Multidetector (64-slice) CT scanning has provided the mainstay in radiological assessment. It has a reported sensitivity of 85% and specificity of 94% for the diagnosis of bladder cancers [11]. Detection is dependent on the morphology and size of the tumor. Flat lesions, carcinoma *in situ* (CIS), tumors less than 1 cm and in those whom recent resection has been performed, are more likely to be falsely negative [5,11,12]. Previous biopsy, inflammation, systemic chemotherapy and intravesical drugs also interfere with interpretation [5,13].

CT remains the modality of choice for investigating hematuria and has replaced intravenous urogram (IVU) at most centers; its precise role in diagnosing bladder cancer is controversial. Although a 64-slice multidetector CT provides high spatial resolution allowing visualization of extravesical spread it is not reliable in determining the extent of locoregional disease [14]. It is limited by interobserver variability and inability to distinguish the muscle layers of the bladder [5,15].

### Ultrasound (US)

US is at present not used routinely in clinical practice for the assessment of known bladder cancer, although the presence of hydronephrosis is suggestive of MIBC. It is however an important diagnostic tool in the investigation of hematuria in particular to assess large renal masses/upper renal tracts. Bladder tumors may be visualized by ultrasound but a negative test does not exclude the presence of bladder cancer.

Two-dimensional transabdominal and transvaginal US have been investigated with regards to aiding bladder cancer staging but transurethral ultrasound has demonstrated the best ability to visualize depth of tumor penetration through the bladder wall [16,17]. Use of two-dimensional US is often limited by the subjectivity and expertise of the examiner. It is also unreliable in determining deeply infiltrating disease and nodal involvement [5]. Contrast-enhanced US (CE US) has been shown to be superior to conventional US in differentiating non-invasive and invasive bladder tumors [18,19]. Prior to TURBT CE US has a reported accuracy of 88.4% versus 72.1% compared to standard two-dimensional US, with 94.7% sensitivity for tumors greater than 5 mm; for lesions less than 5 mm sensitivity is reduced to 20% with a negative predictive value of 28.6% [18].

Three-dimensional US has been developed to provide reconstruction of the actual tumor with visualization of the bladder wall layers. The volume data can be retrieved and manipulated as if in real time, which increases objectivity, allows views in multiple planes to be obtained and improves the rate of primary bladder tumor diagnosis (88.9% with two-dimensional US versus 100% with three-dimensional US) when identifying T3b disease [20]. The disadvantage of this technique is that the entire tumor is not visualized and detection is particularly difficult when flat, plaque like tumors are present, there is coexistent calcification, the abdominal wall is rigid or the patient has central obesity [21].

Three-dimensional CE US uses enhanced images in three orthogonal planes and reflections of microbubbles to depict blood vessels. This has been shown to be clinically useful in differentiating MIBC and NMIBC [21]. Three-dimensional CE US may also have future role in assessing treatment response of the primary tumor to guide bladder-sparing approaches. Its use in assessing treatment has been evaluated other solid tumors including primary liver cancer following local therapy [22].

Although recent developments in US techniques overcome many of the limitations of two-dimensional US, they remain under investigation and have yet not translated in to widespread clinical use.

### Magnetic resonance imaging (MRI)

MRI has excellent soft tissue resolution and multiplanar capabilities, which has made it an important staging modality for bladder cancer. Fundamental to its importance in local staging is the ability to manipulate image contrast by using different sequences. On T2-weighted images the bladder tumor is usually more conspicuous. The signal from perivesical fat can be suppressed using short tau inversion recovery (STIR) sequences, allowing signal from the tumor to be highlighted by suppressing signal from adjacent surrounding normal tissue [5,23,24].

In conjunction with gadolinium-containing contrast (Gd-Ca), MRI has an accuracy of 85% in differentiating NMIBC from MIBC and 82% accuracy in distinguishing organ confined disease from non-organ confined disease [25]. However, Gd-Ca should be avoided in those with renal impairment (estimated glomerular filtration rate (GFR) <60 ml/minute), as there is an increased risk of developing nephrogenic systemic fibrosis [26].

The clearest advantage of MRI over CT is the ability to determine the presence of muscle-invasive and extravesicle disease, and is our preferred modality of local staging prior to definitive radical treatment in MIBC.

The use of functional MRI imaging to provide biological information of tumor characteristics is under investigation. Dynamic contrast-enhanced MRI (DCE-MRI) enables *in vivo* assessment of tumor blood flow and permeability using paramagnetic contrast agents. Visualization of tumor blood flow can be used to identify areas of hypoxia and subsequently be used to predict treatment response [27]. It is an effective biomarker in predicting pathological complete response in those receiving primary chemotherapy for breast cancer and chemoradiotherapy for rectal cancer [23,28].

Intrinsic-susceptibility-weighted or blood-oxygenation-level-dependent (BOLD) MRI, exploits the difference in magnetic susceptibility of oxyhemoglobin and deoxyhemoglobin. Deoxyhemoglobin is a paramagnetic molecule that allows it to act as an intrinsic contrast agent. BOLD MRI image acquisition during high oxygen concentration inhalation (carbogen, 95% oxygen, 5% carbon dioxide) reflects improved tumor oxygenation and blood flow and may help identify patients more likely to benefit from carbogen radiosensitization [29].

Diffusion-weighted MRI (DW MRI) is a functional imaging technique dependent on the inhibitory effect of cell membranes to the random motion of water molecules (Brownian motion) to generate image contrast by applying two equally sized but opposite diffusion sensitizing gradients, characterized by their b-values. As tumors have greater cellularity than normal tissue they demonstrate higher signal intensity (that is, restricted diffusion on MRI, reflected in the low mean apparent diffusion coefficient value (ADC)). This has the potential to provide both qualitative and quantitative information to aid tumor assessment. Histogram analysis of ADC through the entire tumor volume captures the diffusivity microenvironment and may aid identification of the heterogeneity known to exist within tumors that may have prognostic and predictive value [30-34].

DW MRI is also more accurate than T2-weighted MRI in staging both organ confined (≤pT2) (69.7% versus 15.1%) and higher stage tumors (92.5% versus 80.1%), with a reported sensitivity of 98.1% and positive predictive value of 100% [35]. There is also evidence that the ADC value may help identify high-grade tumors, with low ADC (<1 × 10^-3^ mm^2^/s) suggesting G3 disease [36].

Following treatment the ADC value increases, reflecting decreased cellularity, consistent with response. In other tumor types change in ADC has been used to identify and quantify early treatment response that may occur before conventional assessment of response is seen (for example, the Response Evaluation Criteria In Solid Tumors (RECIST) criteria), or where evaluation of morphological change is difficult to interpret [34,37-39]. DW MRI therefore has the potential for monitoring treatment response to chemotherapy or radiotherapy with identification of early non-responders who may benefit from change in treatment approach.

Conventional assessment of local response is with cystoscopy. One study has explored the role of DW MRI to assess response to chemoradiotherapy in 23 patients with MIBC. It demonstrated that the ADC was the only significant, independent predictor of chemoradiotherapy response with a sensitivity, specificity and accuracy of 92%, 90% and 91% respectively. Consistent with other studies higher ADC was associated with unfavorable response [40].

Preliminary results for lymph node evaluation using DW MRI do not appear to be accurate (sensitivity 76.4%, specificity 89.4%, positive predictive value 86.6% and negative predictive value 71.4%) [41].

The clinical use of functional MRI in bladder cancer assessment is not yet clearly defined but work to date suggests it likely to provide important information that may help guide treatment selection.

### Nanoparticle-enhanced MRI

Involved lymph node detection by convention is governed by size and shape. Nodes greater than 1 cm are considered malignant on CT with a sensitivity of 85%, specificity of 67%, with a false negative rate of 21% [5,14]. However, enlarged reactive, non-malignant lymph nodes can mimic metastatic involvement with these modalities. Techniques that evaluate nodal function rather than morphology are aimed to more accurately characterize nodal disease.

Lymphotropic nanoparticle enhanced MRI exploits nodal macrophage function to detect metastases using ultra-small super-paramagnetic particles of iron oxide (USPIO) (ferumoxtran-10, Sinerem®). After intravenous administration, the USPIO particles reach the lymph nodes via the lymphatics. Benign nodes have functioning macrophages, which phagocytose the USPIO causing them to accumulate within the node. This causes a drop in signal intensity on T2-weighted images. Metastatic lymph nodes that are partially or completely infiltrated are unable to take up these particles effectively. These nodal regions retain their signal intensity on T2-weighted images allowing detection on post-contrast imaging [42,43].

Nodal enhancement is dependent on the tumor burden. Failure to detect microscopic foci of metastatic disease in very small lymph nodes leads to false negative results. False positives are due to reactive hyperplasia, localized nodal lipomatosis and insufficient USPIO [24]. Despite these limitations, the reported accuracy when evaluated prospectively in 58 patients prior to surgery is 95%, with sensitivity of 96%, specificity of 95%, positive predictive value of 89% and a negative predictive value of 98% [44]. However, USPIO are no longer readily available, which limits the scope for further investigation and clinical application.

### Virtual cystoscopy (VC)

Three-dimensional surface modeling is possible using cross sectional data obtained from CT or MRI, allowing indirect visualization of the mucosa and simulation of endoscopic evaluation. This method has been used to assess a number of other hollow organs including the colon and bronchus. Once source images are obtained, VC is performed on a dedicated workstation using a variety of computer algorithms [45-48].

Previous VC studies have focused on the potential diagnostic capability of evaluating hematuria. In a meta-analysis of 3084 patients from 26 studies to determine the validity of VC by CT, MRI or US, the pooled sensitivity for bladder cancer detection using CT virtual cystoscopy, magnetic resonance virtual cystoscopy and US was 93.9%, 90.8% and 77.9% respectively. The pooled specificity for bladder cancer detection was 98.1%, 94.8% and 96.2% respectively [49].

**Figure 1:**
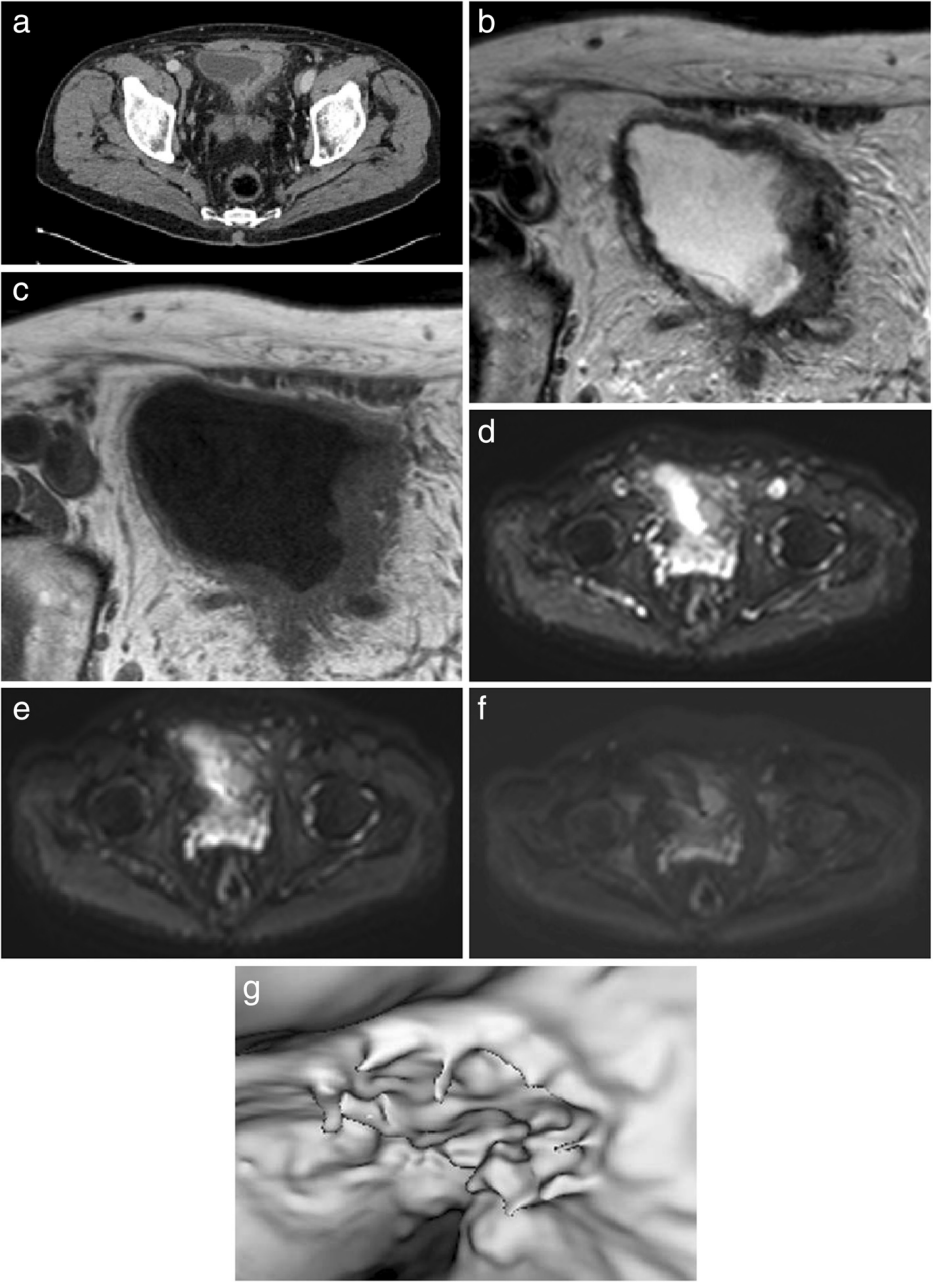
**Patient with known T2 N0 M0 bladder cancer (left bladder wall):** (**a**) contrast-enhanced computed tomography (CT) scan, (**b**) axial T2-weighted image performed on a 3 T magnetic resonance imaging (MRI) unit showing a hypointense lesion, (**c**) corresponding T1 image, (**d**) axial diffusion-weighted (DW) MRI at b-value 0, (**e**) axial DW MRI at b-value 100, (**f**) axial DW MRI at b-value 750, and (**g**) magnetic resonance virtual cystoscopy (MRVC) of same tumor with three-dimensional reconstruction of tumor bed showing opening into adjacent diverticulum.

The advantage of this technique is that it is non-invasive, making it a potential alternative for those unable to tolerate conventional cystoscopy. It also allows visualization of areas that are difficult to access such as the bladder neck and mucosa within diverticulae. The disadvantages include inability to obtain pathology and low sensitivity in identifying smaller tumors (<1 cm), flat lesions and CIS [24,46]. Although VC is unlikely at present to replace conventional cystoscopy it may be considered in conjunction allowing the possibility of minimally invasive follow-up. Figure 1 illustrates appearance of tumor as seen on VC and on CT and MRI.

### Positron emission tomography (PET)

#### PET/CT: metabolic tracers

Composite PET/CT images provide three-dimensional whole body structural and functional information. The patient first moves through a spiral CT then a gamma camera in a single investigation. Radiotracers are used to identify the altered metabolic activity occurring within tumors. Metabolic change detectable by PET may precede anatomical changes on CT or MRI leading to greater sensitivity as compared to conventional axial imaging alone. This uptake is quantified using the standardized uptake value (SUV). Uniform radiotracer distribution throughout the body produces a SUV of 1 [24].

^18^ F-Flurodeoxyglucose (18-FDG) is currently the most commonly used PET tracer in oncological imaging and has an established role in the initial staging, response assessment and recurrence detection of many cancer types [50-53]. Its use is dependent on the increase glucose metabolism occurring within the tumor. However, it cannot distinguish between increased metabolic rate occurring as a result of infection, inflammation or the normal physiological activity in some organs [24].

The use of 18-FDG-PET in staging primary bladder disease, locally recurrent and perivesical nodal disease has been difficult because the interference caused by the urinary excretion of the isotope. A number of techniques encouraging adequate washout of 18-FDG from the urinary tract have been investigated to overcome this. These include elective voiding, catheterization, bladder irrigation, and forced diuresis with intravenous frusemide prior to delayed image acquisition [54-57].

Catheterization and irrigation prior to FDG-PET imaging has a reported 40% false positive rate for detection of recurrent or residual bladder cancer [57]. These measures are invasive, making them less acceptable to patients, and continuous bladder irrigation during image acquisition increases staff exposure to radiation [58].

In those whom FDG-urine washout was encouraged by diuretic injection, oral hydration and voiding, the sensitivity and specificity for FDG-PET CT was 86.7% and 100% respectively for detecting recurrent disease within the bladder [56]. Further investigation is necessary however to evaluate the impact of radiotherapy, endoscopic intervention and intravesical chemotherapy on FDG-PET interpretation within the bladder. When imaging is performed after chemotherapy the sensitivity decreases to 50% and therefore 18-FDG PET results should be interpreted with caution following systemic treatment [59].

In a meta-analysis of the overall diagnostic accuracy of 18-FDG PET in bladder cancer, 6 studies involving 203 patients were assessed. The sensitivity and specificity of 18-FDG PET or PET/CT for staging or restaging (metastatic lesions) of bladder cancer was 82% and 89% respectively. The global measure of accuracy was 0.92 [60]. The limitations accepted by the authors include variation in the imaging technique used, one study used PET alone which meant anatomical accuracy because of the poor spatial resolution was lost, three studies were retrospective in nature and only two studies assessed detection of the primary tumor.

Although there is evidence that FDG PET-CT has a diagnostic role for identifying metastatic bladder disease, in our clinical practice it is not used as principal staging modality because of the limitations discussed above. In certain circumstances, however, it provides important contributory information when CT or MRI alone raises uncertainty regarding staging.

Alternative radiotracers that are dependent on cell proliferation, apoptosis, and angiogenesis, hypoxia and growth factors are also under investigation [61,62]. ^11^C-Choline and ^11^C-methionine are not excreted in the urine and may have role in future imaging of bladder cancer [24,54,63-65]. There is however limited data at present to support routine clinical use.

Choline is an essential component of cell membranes. Malignant tumors have a high turnover of cellular membranes representing their increased proliferation rate [66]. The normal bladder has low uptake with ^11^C-choline [63]. In the preoperative staging of 18 patients, ^11^C-choline was highly positive for primary and metastatic bladder cancer. Uptake was seen in all primary transitional cell carcinomas (mean SUV 7.3 ± 3.2 SD). In six patients, ^11^C-choline uptake was seen in lymph nodes as small as 5 mm; of those, four proceeded to surgery and three had pathological conformation of nodal disease [64].

^11^C-Choline has also been used to detect residual disease after TURBT. In a prospective study of 27 patients prior to radical surgery, ^11^C-choline PET was comparable to CT alone for detecting residual cancer after TURBT but appeared to be superior for detecting nodal involvement, with reported sensitivity and specificity of 62.5% and 100% versus 50% and 68.4% for contrast-enhanced CT alone [65].

^11^C-Choline has a short half-life of approximately 20 minutes. Therefore clinical use is restricted predominantly to those centers with an on-site cyclotron. ^18^ F-choline analogs with greater half-lives have been developed to overcome this; however, significant urinary excretion occurs as compared to ^11^C-choline [67]. This represents a disadvantage for pelvic imaging as previously discussed unless adequate urinary washout can be encouraged.

^11^C-Methionine is a radiolabeled amino acid and is a potential tracer for visualizing protein metabolism, cellular proliferation and amino acid transport. Compared to 18-FDG PET in identifying primary tumors within the bladder, uptake is proportional to tumor stage, with a reported sensitivity of 78% in tumors greater than 1 cm but its value in local staging is not superior to conventional imaging [54].

^18^ F Fluoride is a bone-seeking radiopharmaceutical that accumulates at sites of increased bone formation reflecting increased osteoblastic activity occurring within metastases. It has been shown to have increased diagnostic accuracy as compared to technetium-99 m-methylene diphosphonate (99mTc-MDP) planar or single photon emission computed tomography (SPECT) in other solid tumors [68,69].

Non-FDG tracers are not in widespread clinical use partly because of the lack of robust evidence supporting clinical benefit but also because of their cost and limited availability.

#### PET/CT: receptor specific radiopharmaceuticals

Imaging biomarkers using PET-CT and radioimmunotherapy opens the possibility of an individualized therapeutic and imaging approach. The rationale is that a tumor specific target is combined with a therapeutic radioactive agent. The selective accumulation within the target tissue can then be visualized on PET. These imaging techniques have the potential to permit an ‘image and treat approach’ by allowing tumor staging, estimation of radiation dose distribution prior to therapy, and early monitoring of treatment response [70].

Commonly used nuclides in other tumors types include β emitters such as ^131^I and ^90^Y. They are attached to somatostatin receptor binding agents such as ^90^Y-DOTA-d-Phe(1)-Tyr(3)-octreotide (^90^Y-DOTATOC) for the treatment of neuroendocrine tumors and to antibodies in ^131^I-tositumomab (Bexxar®), ^90^Y-rituximab (Zevalin®) to target the CD20 antigen on B cells for the treatment of lymphoma [70,71].

Overexpression and amplification of epidermal growth factor receptor (EGFR) (HER1 or ErdB1) and, or the HER2 gene is found in bladder cancers [72,73]. It therefore represents a potential target for both molecular imaging and therapy in those with known HER2-positive disease [61]. Monoclonal antibodies for example, trastuzumab labeled with ^18^ F, allows *in vivo* monitoring of HER2 expression by PET as well as assessing change in HER2 expression with therapy [74,75]. Trastuzumab has also been labeled with nucleotides suitable for therapy with the future possibility of treating metastatic disease and improving the outcome of those with HER2 bladder cancer [70,76,77].

#### PET/MRI

Image acquisition with PET/CT occurs sequentially rather than simultaneously. This means there is loss of temporal correlation, and additionally from the patient’s perspective scanning time is significantly longer. The possibility of assessing different functional parameters using PET, DW MRI and combining that data with high-resolution anatomical information from MRI may provides new opportunities to study pathological and biochemical processes *in vivo*[78-81].

**Figure 2:**
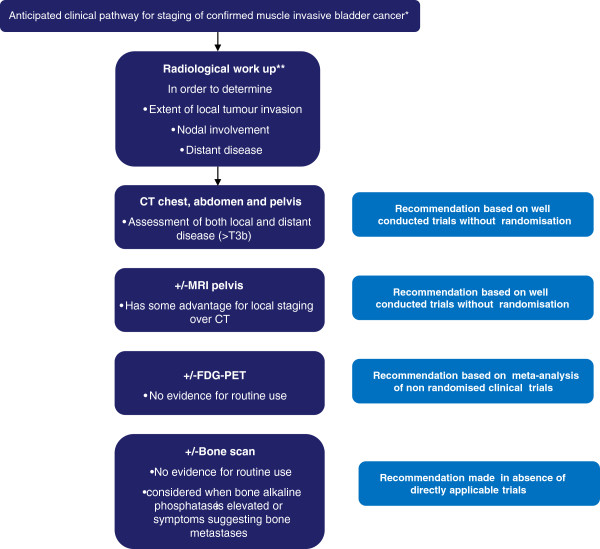
**Anticipated clinical pathway for staging of confirmed muscle invasive bladder cancer.** *Diagnostic investigations for haematuria differ from imaging to determine extent of local and distant disease in confirmed muscle invasive bladder cancer. **Based on European Association of Urology guidelines 2012, available at http://www.uroweb.org/guidelines/online-guidelines/ and http://www.nccn.org/professionals/physician_gls/f_guidelines.asp#site.

Most patients in the UK will present to urology teams via a ‘one-stop hematuria clinic’, where a two-dimensional US, urine cytology and flexible cystoscopy will be carried out. On the basis of these results a TURBT will be performed if appropriate. A CT scan will be undertaken where there is evidence of MIBC, high-grade NMIBC or suspicion of an upper tract lesion. However, in view of the evidence supporting MRI (as discussed above) our preferred practice is to stage local disease using this modality. A summary of imaging modalities and their current clinical role in staging known MIBC is presented in Table 1 and Figure 2.

**Table 1:** A summary of imaging modalities and their current clinical role(s) in staging known muscle-invasive bladder cancer

**Technique**	**Description**
CT	Mainstay of assessment in locally advanced and metastatic disease. Limitations are in assessing primary tumor.
US	Two-dimensional techniques have no role in routine assessment of primary tumor, however presence of hydronephrosis is suggestive of MIBC. Contrast-enhanced and three-dimensional techniques are under investigation.
MRI	Useful in identifying muscle-invasive and extravesical disease. Functional MRI currently under investigation as a predictive tumor biomarker.
Virtual cystoscopy	Both CT and MRI data can be used to reconstruct bladder mucosa and simulate endoscopic evaluation. Unlike for other tumor sites such as GI, it is not used routinely.
PET-CT	FDG-PET-CT not used as initial staging modality. Often used in conjunction with other imaging if uncertainly exists. FDG use for staging local disease limited predominantly by urinary excretion. Alternative isotopes and receptor specific molecules are under investigation.

CT, computed tomography; FDG, flurodeoxyglucose; GI, gastrointestinal; MIBC, muscle-invasive bladder cancer; MRI, magnetic resonance imaging; PET, positron emission tomography; US, ultrasound.

### Patient perspectives

Image acquisition time and tolerability of any proposed scan is important. CT image acquisition is usually within minutes but some research MRI protocols may take up to 1 h. This can impact on patient compliance and predispose results to motion artifacts.

Use of MRI and US are free from ionizing radiation as compared to CT and PET/CT. At our institution a 64-slice multidetector CT scan of the chest, abdomen and pelvis is associated with radiation exposure of 16 mSv during imaging; PET/CT is associated with exposure of 14 mSv (PET component 8 mSv; 6 mSv from a rapid image acquisition CT). The clinical significance of these values in terms of inducing second malignancy is small in the context of the overall poor prognosis from muscle-invasive and metastatic bladder cancer at present. MRI scanning also does not require the use of iodine contrast agents that can induce reactions potentially anaphylactic in some individuals. In addition to allergy, intravenous contrast is also omitted for CT scanning in the presence of significant renal impairment.

Technology is rapidly changing so the ability to use this information to identify the most effective intervention for patients is critical. In the future we anticipate it will be routine to tailor a patient’s treatment plan to both the physiological and physical characteristics of their disease, to monitor effectiveness of the intervention allowing a more dynamic approach to treatment.

## Conclusions

Accurate staging is important in determining prognostic information and identifying appropriate treatment options. CT and MRI remain central to bladder cancer staging. The role of PET-CT using current tracers in staging and guiding management of bladder cancer remains to be defined. Future developments in functional imaging are likely to be important in predicting treatment response allowing timely identification of non-responders to guide appropriate change in treatment but further studies are required to determine which techniques or combination of techniques will optimize patient care.

## Competing interests

The authors declare that they have no competing interests.

## Authors’ contributions

SH drafted the manuscript. SH and RH revised the manuscript. Both authors read and approved the final version of the manuscript.

## Authors’ information

RH is a Reader at the Institute of Cancer Research (ICR), leading a team in the Division of Radiotherapy and Imaging that researches bladder and testicular cancer. He is also an Honorary Consultant in Urological Oncology at The Royal Marsden Hospital, where he manages and treats patients with urological cancer. SH is his Research Fellow at the ICR; together they are investigating image-guided (IGRT) and intensity-modulated radiotherapy (IMRT) for the treatment of muscle-invasive bladder cancer and the role that functional imaging plays in its management.

## Pre-publication history

The pre-publication history for this paper can be accessed here:

http://www.biomedcentral.com/1741-7015/11/104/prepub
